# Integrating Functional Consequence Annotation With *PAH* Allelic Phenotype Values Refines Prediction of Tetrahydrobiopterin Responsiveness

**DOI:** 10.1002/jimd.70220

**Published:** 2026-06-21

**Authors:** Nastassja Himmelreich, Nenad Blau

**Affiliations:** ^1^ Dietmar‐Hopp Metabolic Center and Centre for Pediatrics and Adolescent Medicine University Children's Hospital Heidelberg Germany; ^2^ Zentrum für Humangenetik Tübingen Tübingen Germany; ^3^ Divisions of Metabolism University Children's Hospital Zürich Switzerland

**Keywords:** allelic phenotype value, genotype‐based prediction, PAH, phenylketonuria, sapropterin, tetrahydrobiopterin

## Abstract

Tetrahydrobiopterin (BH4; sapropterin) responsiveness in phenylalanine hydroxylase (PAH) deficiency is genotype dependent, yet many patients remain untested. Allelic phenotype values (APV) summarize allele severity, but responsiveness can be heterogeneous within APV strata. We assessed whether integrating functional consequence annotation from Ensembl variant effect predictor (VEP) improves genotype‐based prediction of BH4 response. We analyzed 23 640 individuals with biallelic *PAH* genotypes and BH4 status (RESP, S‐RESP, N‐RESP, or not tested). Tested individuals were used for model development (responders defined as RESP+S‐RESP; nonresponders as N‐RESP). APV values were assigned from published APV resources and merged at the variant level. Functional consequence predictors were derived from VEP output and included strict predicted loss‐of‐function (pLoF) flags, splice‐impact scores (SpliceAI maximum delta score), and missense pathogenicity predictions (SIFT and PolyPhen). Genotype predictors were constructed using a milder‐versus‐severer allele framework, with the milder allele defined as the allele with the higher APV. Models were evaluated using genotype‐held‐out cross‐validation (GroupKFold by genotype). Among 4640 tested individuals, 2044 (44.1%) were BH4 responders. Responder rates were enriched in milder phenotypes and increased monotonically across milder‐allele APV bins. In genotype‐held‐out evaluation, integrating functional consequence predictors with APV improved discrimination modestly overall and more clearly in intermediate APV genotypes. Genotype predicts BH4 responsiveness with high performance under stringent genotype‐held‐out validation, and VEP‐derived functional consequence annotation provides modest complementary value beyond APV, particularly for intermediate‐severity genotypes.

## 1 Introduction

Phenylalanine hydroxylase (PAH) deficiency is the most common cause of hyperphenylalaninemia and phenylketonuria (PKU), with clinical severity mostly determined by residual PAH activity and resulting blood phenylalanine (Phe) concentrations [1], as well as potential epigenetic factors. Since the introduction of newborn screening, early dietary treatment has substantially improved neurocognitive outcomes; however, lifelong dietary restriction remains burdensome and adherence may be difficult, particularly in adolescence and adulthood [2]. Pharmacologic options that increase residual PAH activity can reduce dietary burden and improve metabolic control in selected patients [3].

The first description of BH4‐responsive PAH deficiency demonstrated that BH4 administration can lower blood Phe in some patients, indicating that responsiveness is biologically plausible and clinically meaningful [4]. Subsequent clinical studies established BH4 (sapropterin dihydrochloride) as an effective treatment alternative or adjunct to dietary therapy, particularly in milder forms of PKU [5], and helped define operational criteria for responsiveness. A widely used definition is a reduction of blood Phe of at least 30% following BH4 loading or short‐term treatment [6], although responsiveness is best viewed as a continuum that depends on the testing protocol and baseline metabolic state. Mechanistically, BH4 responsiveness is generally associated with *PAH* variants that preserve residual activity and/or protein stability, consistent with the concept that sapropterin can act as a pharmacologic chaperone in addition to serving as a cofactor (dual mode of action) [7]. Best‐practice recommendations have emphasized standardized testing strategies, as well as their interpretation, and follow‐up to support evidence‐based use of sapropterin in clinical care [8].

Because BH4 responsiveness is strongly influenced by genotype, multiple approaches have been proposed to translate *PAH* variant information into clinically actionable predictions. The allelic phenotype value (APV) framework provides a quantitative, allele‐level severity scale derived from curated genotype–phenotype associations and has been used to predict metabolic phenotype and inform expectations regarding BH4 responsiveness [9]. Large international datasets have further expanded the catalog of *PAH* variants and genotypes, enabling more robust estimates of variant severity and population‐level genotype distributions and reinforcing the importance of the higher‐residual‐activity allele for clinically relevant outcomes [10]. Nevertheless, genotype‐based prediction of BH4 responsiveness remains imperfect in practice, particularly for intermediate‐severity genotypes and for variants with limited empirical data.

Functional consequence annotation provides a complementary, biologically informed source of information that may refine genotype‐based prediction [7, 8]. Integrating such functional signals with established APV‐based severity metrics may be particularly useful where APV alone leaves substantial heterogeneity, such as in intermediate APV strata or for variants with limited clinical classification [9, 10].

Despite established protocols for BH4 testing, many patients with PAH deficiency remain untested, and genotype‐based prediction of BH4 responsiveness is still incomplete, particularly for rare or previously uncharacterized variants. In this study, we used the BIOPKU resource to investigate how *PAH* genotype relates to BH4 responsiveness in a cohort of 23 640 individuals, including 4640 with recorded BH4 testing results. We aimed to determine whether integrating established allele‐severity information with standardized functional variant annotation improves prediction of BH4 response and provides a practical framework for prioritizing BH4 testing and treatment decisions.

## 2 Methods

### 2.1 Study Design, Setting, and Outcome Definition

This was a retrospective observational study using genotype and BH4 response data curated in the BIOPKU database. The primary objective was to investigate genotype determinants of BH4 responsiveness and to assess whether integrating functional consequence annotation with allelic phenotype values (APV) improves genotype‐based prediction of BH4 response.

BH4 response categories (RESP, S‐RESP, N‐RESP, and NT) were retrieved from the BIOPKU database and reflect the clinical interpretation of sapropterin (BH4) responsiveness testing performed at contributing centers. RESP indicates responders with a documented clinically meaningful reduction in blood phenylalanine after BH4 administration; S‐RESP indicates slow responders with a delayed but clinically meaningful reduction; N‐RESP indicates nonresponders without a clinically meaningful reduction under the applied protocol; and NT indicates that no BH4 responsiveness test result was available or recorded. For prediction analyses, RESP and S‐RESP were grouped as “responders” and compared with N‐RESP as “nonresponders,” consistent with international guidance for the evaluation of BH4 responsiveness [8]. Where available, this interpretation was typically based on established criteria such as a ≥ 30% reduction in blood phenylalanine following BH4 loading or short‐term treatment, while acknowledging protocol‐dependent variation.

Secondary endpoints included BH4 responder proportions across metabolic phenotype categories, APV strata, APV‐defined 0‐allele burden, genotype functional classes, and variant‐level summaries when a given allele represented the milder allele within a genotype.

### 2.2 Data Sources and Cohort Definition

Genotype and BH4 response data (two *PAH* alleles recorded as HGVS cDNA nomenclature; cDNA1 and cDNA2), metabolic phenotype category, BH4 response status, and blood phenylalanine concentrations where available were retrieved from the BIOPKU database. Variant‐level APV and functional consequence annotations used for feature construction were obtained from BIOPKU exports. APV in BIOPKU follows the published APV framework [9, 10]. Functional consequences and in silico prediction features in BIOPKU were generated using Ensembl variant effect predictor (VEP) and were parsed for downstream analyses.

### 2.3 APV Assignment and Higher‐Residual‐Activity Allele Framework

APV were assigned to *PAH* variants using published APV resources [9, 10]. APV is a quantitative allele‐level severity score that describes the association of an individual *PAH* variant with metabolic phenotype in PKU. It is derived primarily from phenotypes observed when a variant occurs in a functionally hemizygous state, that is, in trans with an inactive/null allele, so that the clinical phenotype largely reflects the residual effect of the variant under evaluation. APV ranges from 0 to 10, with lower values indicating more severe alleles and higher values indicating milder alleles.

For each individual, the “milder” allele was defined as the allele with the higher APV, that is, the allele predicted to retain higher residual PAH function; the other allele was designated the “severer” allele. This definition follows an enzymatic‐threshold model typical of autosomal recessive enzymopathies, in which total residual activity in compound heterozygotes is largely determined by the allele with greater remaining function. Genotype‐level predictors were then constructed as milder APV and severer APV. In addition, the number of APV‐defined null alleles was calculated per genotype (n0_alleles = 0, 1, or 2), where APV = 0 indicates no residual enzymatic activity. This higher‐residual‐activity allele representation was used consistently throughout the descriptive analyses and predictive modeling.

### 2.4 Functional Consequence Annotation (VEP‐Derived Predictors)

Functional consequence annotation was performed using the Ensembl VEP, applying standard consequence terms and HGVS‐compliant variant representations. For each unique *PAH* variant, VEP outputs were used to derive (i) a most severe consequence category and (ii) a standardized set of variant‐level functional predictors.

A strict predicted loss‐of‐function (pLoF) indicator was defined for variants annotated by VEP as stop_variant, frameshift_variant, splice_donor_variant, splice_acceptor_variant, or start_lost. Formally, pLoF(v) was defined as 1 when the most severe consequence of variant v belonged to this set, and 0 otherwise. To capture splice impact beyond consequence labels, splice predictions were incorporated using SpliceAI delta scores reported by VEP (acceptor gain/loss and donor gain/loss). A single splice‐impact feature was defined as the maximum SpliceAI delta score across the four categories, SpliceAI_max(v) = max(ΔS_AG, ΔS_AL, ΔS_DG, ΔS_DL), where each ΔS ranges from 0 to 1.

For missense pathogenicity prediction, SIFT and PolyPhen‐2 values were extracted as numeric scores from VEP output when available and used as continuous predictors. Lower SIFT values indicate greater predicted deleteriousness, whereas higher PolyPhen‐2 values indicate greater predicted damaging effect. Not all predictors are applicable to all consequence classes; for example, SIFT and PolyPhen‐2 are defined primarily for missense variants, whereas SpliceAI scores are most relevant for variants near splice junctions. Non‐applicable or unavailable fields were therefore retained as missing values and handled in the modeling pipeline by fold‐wise median imputation fitted on the training data only.

For genotype‐level modeling, each individual i carried two alleles, v_i1 and v_i2. Variant‐level predictors were mapped to both alleles and then summarized using the milder‐versus‐severer allele representation. Specifically, the “milder” allele was defined as the allele with the higher APV, that is, the allele expected to retain higher residual PAH function, and the other allele was designated the “severer” allele. Allele‐specific annotation features were then assigned accordingly (e.g., milder SIFT, milder SpliceAI_max, milder pLoF, and corresponding severer‐allele features). Annotation was performed on normalized HGVS cDNA variant strings; where multiple transcript‐specific annotations existed, the most severe consequence was used for feature construction.

### 2.5 Statistical Analysis and Prediction Modeling

All analyses were performed in Python (version 3.11). Data processing used pandas (v2.x) and NumPy (v1.x). Prediction modeling and cross‐validation used scikit‐learn (v1.x), including GroupKFold for genotype‐held‐out evaluation, logistic regression, and ROC and precision–recall analyses. Figures were generated using Matplotlib (v3.x), and publication‐ready tables were generated using openpyxl.

BH4 responsiveness was analyzed among BH4‐tested individuals using the BIOPKU classification as the reference outcome. For prediction analyses, responders were defined as RESP or S‐RESP (positive class) and nonresponders as N‐RESP (negative class); individuals classified as NT (not tested) were excluded from model training and performance evaluation and were reserved for downstream application.

Genotype‐based predictors were constructed from (i) allele severity values and (ii) functional consequence annotations. Allele severity was represented using published APV. For each individual, the “milder” (higher‐residual‐activity) allele was defined as the allele with the higher APV, that is, the allele expected to retain higher residual PAH function under an enzymatic‐threshold model typical of autosomal recessive enzymopathies; the other allele was designated the “severer” allele. Genotype predictors included milder_APV, severer_APV, and APV‐defined 0–allele burden (n0_alleles). Functional consequence predictors were derived from Ensembl VEP outputs and included strict pLoF status, splice‐impact prediction summarized as the maximum SpliceAI delta score, and missense pathogenicity scores (SIFT and PolyPhen‐2). These functional predictors were mapped to the two alleles and summarized using the same milder‐versus‐severer allele representation (milder and severer allele features).

To assess generalizability to previously unseen genotypes, models were evaluated using 5‐fold genotype‐held‐out cross‐validation (GroupKFold grouped by unordered genotype), ensuring that all individuals with the same genotype were assigned to the same fold and were therefore either entirely in the training set or entirely in the held‐out test set. Logistic regression was used as the primary classifier. Missing predictor values, which predominantly reflected non‐applicability or unavailability of specific annotation fields for certain variant classes, were retained as missing and handled within each cross‐validation training fold using median imputation fitted on the training data only; the imputation parameters were then applied to the corresponding held‐out fold to prevent information leakage. Continuous predictors were standardized (*z*‐scored) within each training fold and applied to held‐out data using training‐derived parameters to improve numerical stability.

Model performance was quantified using AUROC and precision–recall AUC, computed from pooled out‐of‐fold predicted probabilities across the five folds. For clinically interpretable classification, accuracy, sensitivity, and specificity were calculated at a prespecified probability threshold of 0.5, and confusion matrices were generated from aggregated held‐out predictions.

## 3 Results

### 3.1 Cohort and BH4 Testing

BH4 responsiveness was recorded for 4640 individuals (tested subset; 19.6%): 2044 responders (RESP/S‐RESP; 44.1%) and 2596 nonresponders (N‐RESP; 55.9%). The remaining individuals were NT and were not used for model fitting.

### 3.2 BH4 Responsiveness Stratifies by Metabolic Phenotype and Allele Severity

**FIGURE 1:**
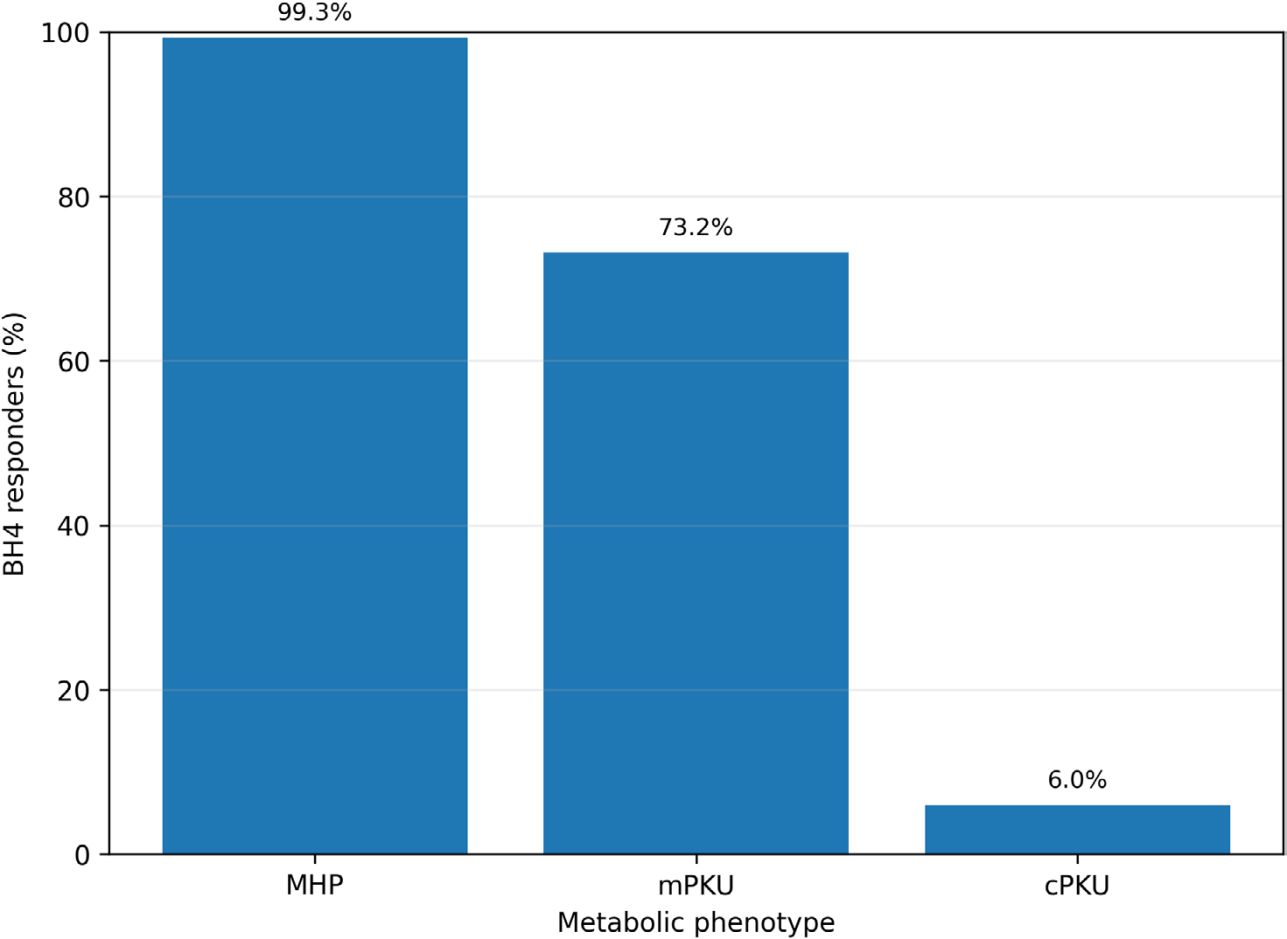
BH4 responder rate by metabolic phenotype. Responder rate (RESP+S‐RESP) among tested individuals stratified by phenotype. Error bars show 95% confidence intervals for proportions. BH4, tetrahydrobiopterin; cPKU, classic phenylketonuria; MHP, mild hyperphenylalaninemia; mPKU, mild phenylketonuria; N‐RESP, nonresponder; RESP, responder; S‐RESP, slow responder.

**FIGURE 2:**
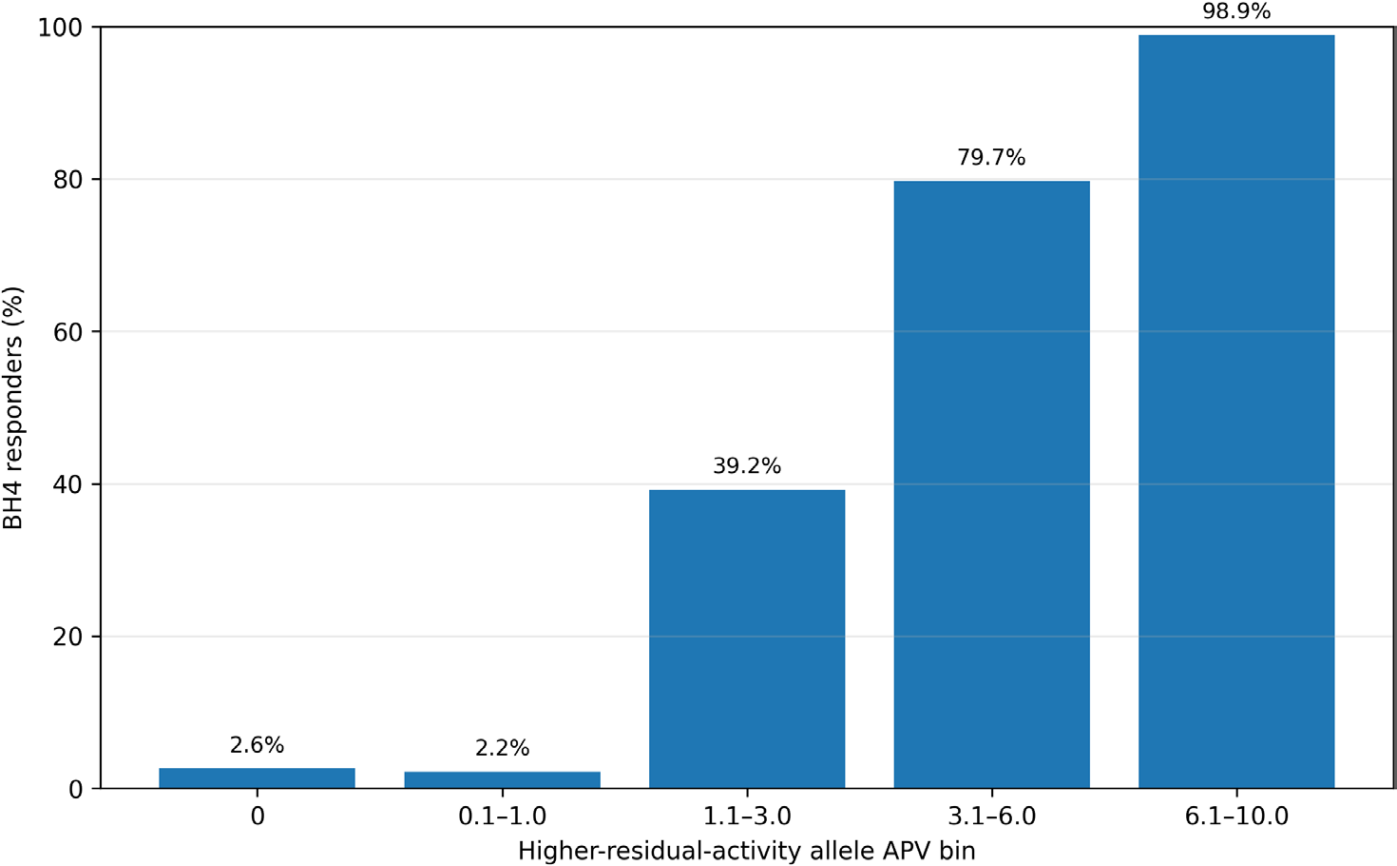
BH4 responder rate by milder‐allele APV bin. Responder rate (RESP+S‐RESP) among tested individuals grouped by the allelic phenotype value (APV) of the milder allele (defined as the allele with higher APV within each genotype). Error bars show 95% confidence intervals. APV, allelic phenotype value; BH4, tetrahydrobiopterin; N‐RESP, nonresponder; RESP, responder; S‐RESP, slow responder.

BH4 responsiveness differed markedly across metabolic categories. Among BH4‐tested individuals, the proportion classified as responders (RESP or S‐RESP) was highest in mild hyperphenylalaninemia (MHP: 692/697; 99.3%), intermediate in mild PKU (mPKU: 1210/1652; 73.2%), and lowest in classic PKU (cPKU: 136/2272; 6.0%) (Figure 1; Table 1). When genotypes were summarized by allele severity, the probability of BH4 responsiveness increased monotonically as the less severe allele became milder (higher APV) (APV: 0: 17/650 (2.6%); 0.1–1.0: 22/1020 (2.2%); 1.1–3.0: 492/1256 (39.2%); 3.1–6.0: 695/872 (79.7%); 6.1–10.0: 811/820 (98.9%)) (Figure 2 and Table 2).

**TABLE 1:** BH4 responsiveness by metabolic phenotype (tested individuals).

Phenotype	Tested (*n*)	Responder (*n*)	Nonresponder (*n*)	Responder rate (%)
MHP	697	692	5	99.3
mPKU	1652	1210	442	73.2
cPKU	2272	136	2136	6

**TABLE 2:** BH4 responsiveness by milder‐allele APV bin (tested individuals).

Milder allele APV	Tested (*n*)	Responder (*n*)	Nonresponder (*n*)	Responder rate (%)
0	650	17	633	2.6
0.1–1.0	1020	22	998	2.2
1.1–3.0	1256	492	764	39.2
3.1–6.0	872	695	177	79.7
6.1–10.0	820	811	9	98.9

### 3.3 APV‐Defined 0‐Allele Burden Is Inversely Associated With BH4 Responsiveness

**FIGURE 3:**
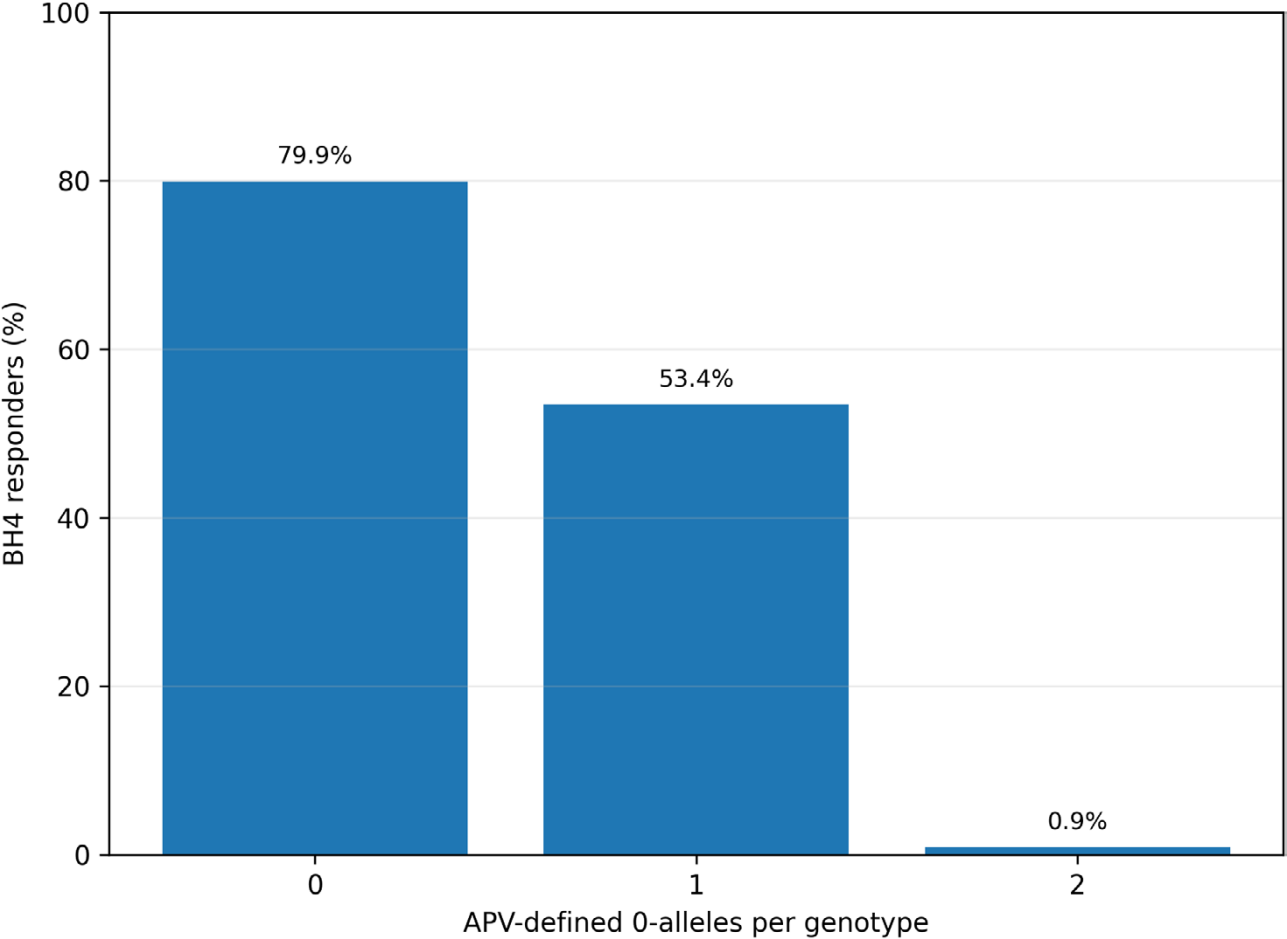
BH4 responder rate by APV‐defined 0‐allele burden. Responder rate (RESP+S‐RESP) among tested individuals stratified by the number of APV‐defined 0 alleles in the genotype (0, 1, or 2). APV, allelic phenotype value; BH4, tetrahydrobiopterin; N‐RESP, nonresponder; RESP, responder; S‐RESP, slow responder.

Increasing APV‐defined 0‐allele burden was associated with lower BH4 responder proportions (Figure 3). Genotypes with no APV‐defined 0 alleles showed the highest responder proportion, whereas genotypes with one or two 0 alleles were progressively less likely to be classified as BH4 responders.

### 3.4 BH4 Responsiveness Differs Across Genotype Functional Classes

**FIGURE 4:**
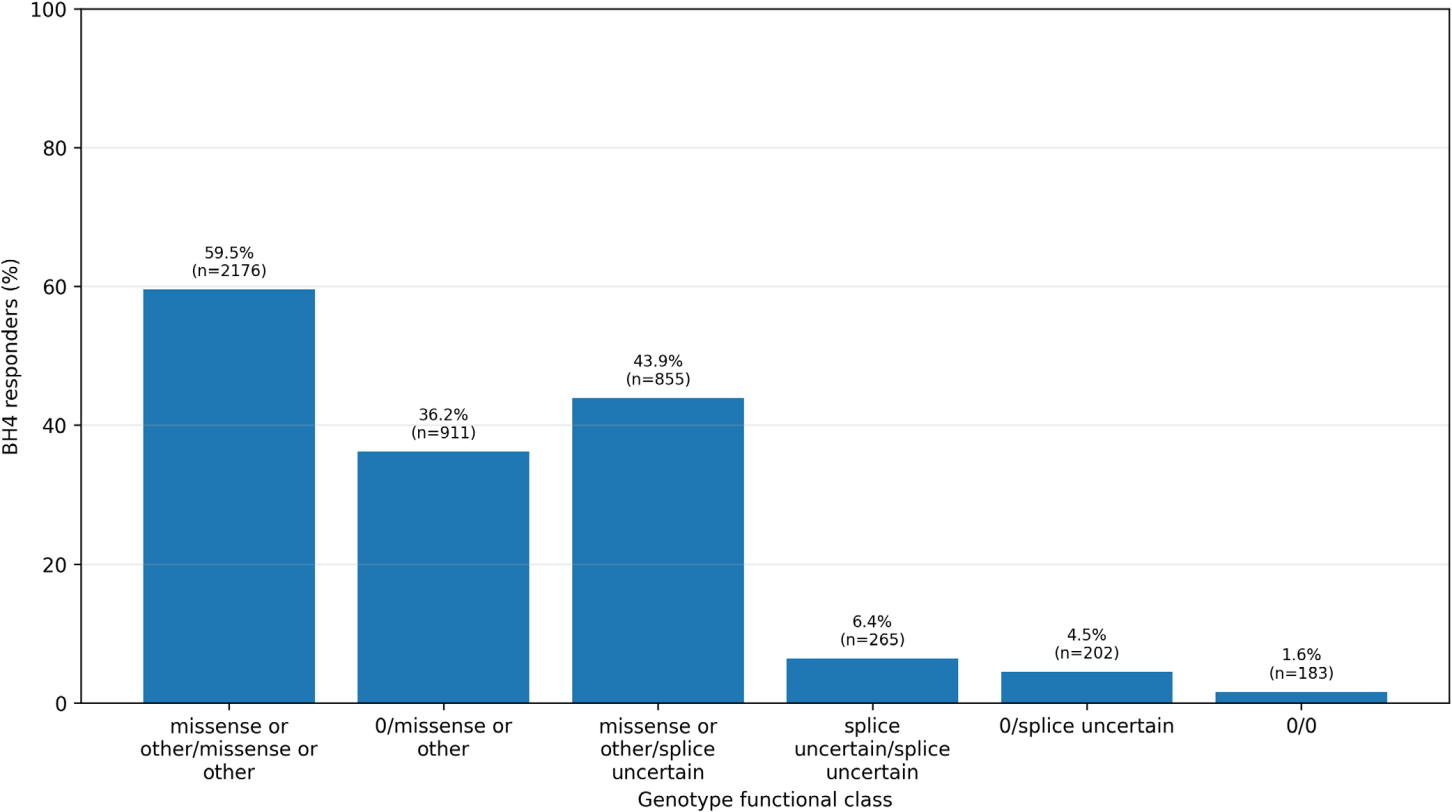
BH4 responder proportion by genotype functional class. Proportion classified as BH4 responders (RESP+S‐RESP) among tested individuals stratified by VEP‐derived genotype functional class. Bars are labeled with responder proportion (%) and the number of tested individuals in each class. BH4, tetrahydrobiopterin; RESP, responder; S‐RESP, slow responder; VEP, variant effect predictor.

BH4 responder proportions also differed across VEP‐derived genotype functional classes (Figure 4). Genotypes composed of two missense‐or‐other alleles showed the highest responder proportion, whereas genotypes containing canonical splice‐disrupting or two pLoF alleles showed markedly lower responder proportions.

### 3.5 Integrating Functional Consequence Annotation Improves Prediction Beyond APV

**FIGURE 5:**
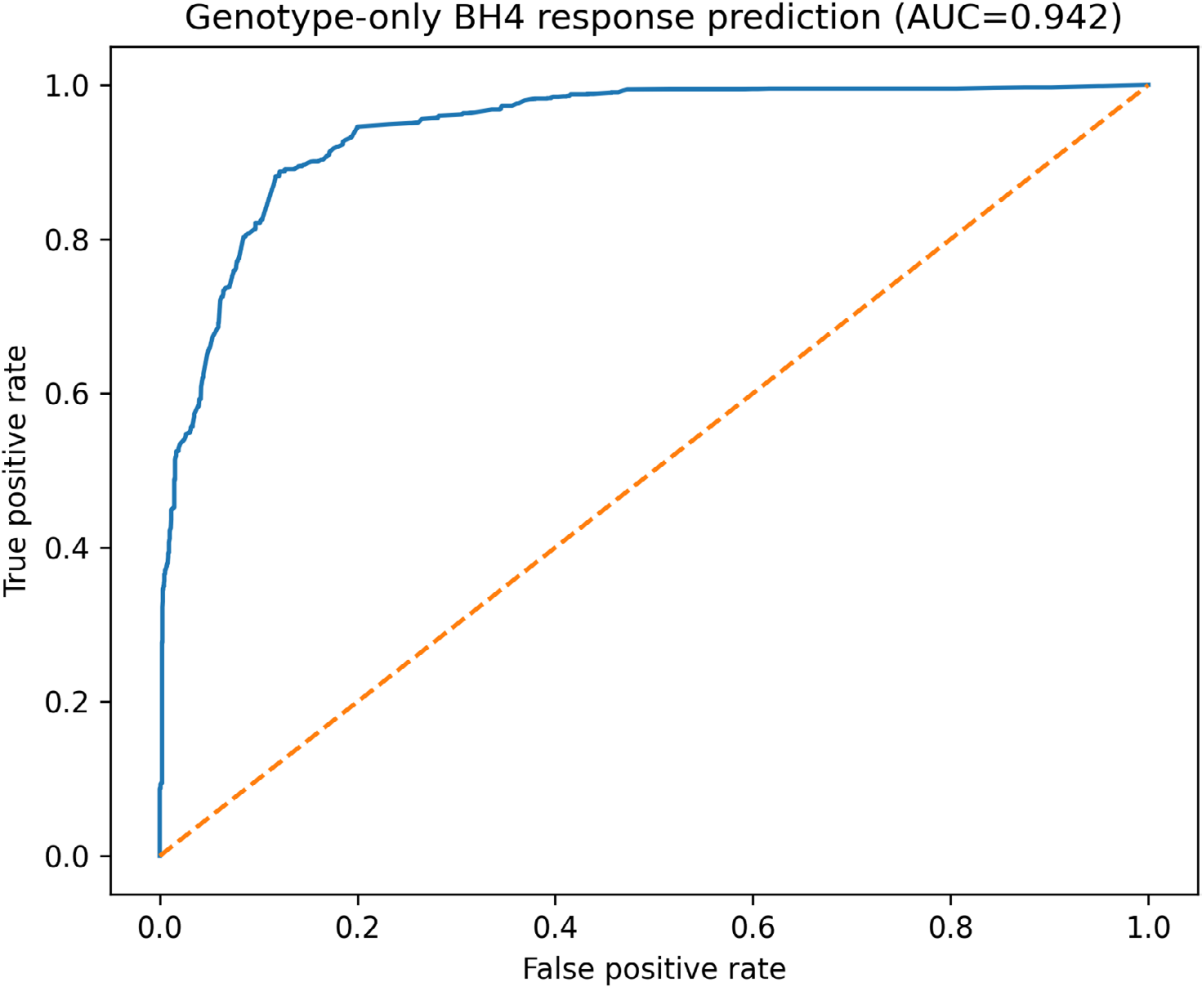
Genotype‐only prediction of BH4 responsiveness. Receiver operating characteristic (ROC) curve for the APV‐only genotype model evaluated by 5‐fold genotype‐held‐out cross‐validation (GroupKFold by genotype). The model used genotype‐derived predictors only, without additional VEP‐based functional consequence features. The area under the ROC curve (AUROC) was 0.942. APV, allelic phenotype value; AUROC, area under the receiver operating characteristic curve; BH4, tetrahydrobiopterin; ROC, receiver operating characteristic.

In genotype‐held‐out cross‐validation (GroupKFold by genotype; *n* = 4618 tested), an APV‐only model discriminated responders from nonresponders well (AUROC 0.942; Figure 5 and Figure S1). Adding VEP‐derived functional consequence predictors, including strict pLoF flags, splice donor/acceptor disruption, and missense pathogenicity scores mapped to the milder and severer alleles yielded only a small incremental increase in discrimination compared with the APV‐only model (ΔAUROC≈0.005) (Figure 5 and Table 3). Given the small magnitude of this difference, it should be interpreted cautiously and not taken, by itself, as evidence of major clinical benefit.

**TABLE 3:** Model comparison for predicting BH4 responsiveness (genotype‐held‐out cross‐validation).

Model	AUC genotype heldout	ΔAUC vs. APV only
APV only (milder+severer APV)	0.945	0
APV+0‐allele count	0.948	0.003
APV+missense functional scores (PolyPhen/SIFT)	0.949	0.004
APV+full functional consequence annotation (pLoF/spliceAI+PolyPhen/SIFT)	0.95	0.005

*Note:* Genotype‐held‐out (GroupKFold by genotype, 5‐fold) AUC for BH4 responsiveness prediction comparing APV‐only vs. APV plus functional consequence annotation derived from VEP (PolyPhen/SIFT; spliceAI; strict pLoF).

### 3.6 Variant‐Level Associations When Variants Act as the Milder Allele

**FIGURE 6:**
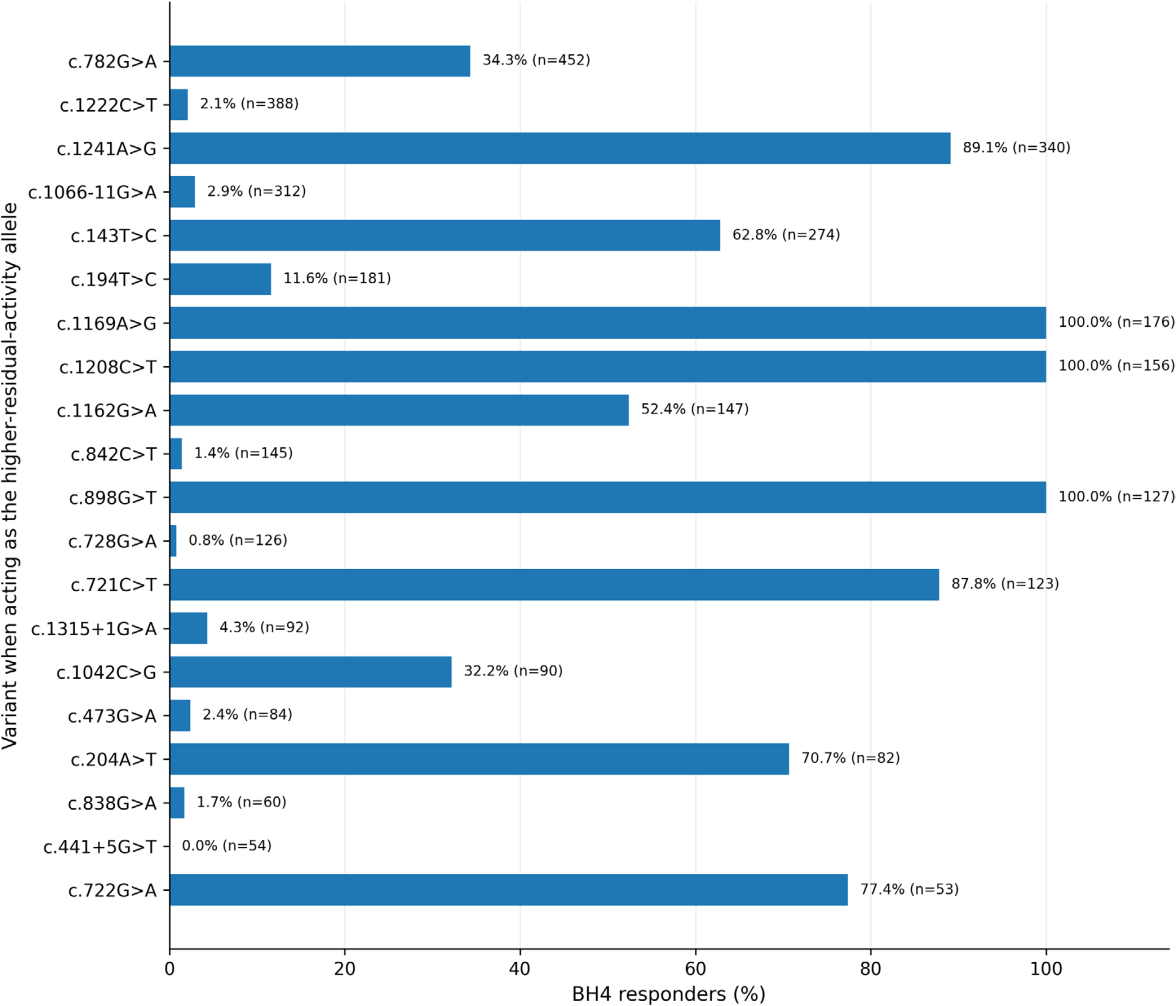
Top 20 *PAH* variants by BH4 responder proportion when acting as the milder allele. BH4 responder proportion (RESP+S‐RESP) for the 20 most frequent *PAH* variants when acting as the milder allele within a genotype, ranked by number of tested carriers. The milder allele was defined as the allele with the higher APV. Bars are labeled with responder proportion (%) and number of tested carriers. APV, allelic phenotype values; BH4, tetrahydrobiopterin; PAH, phenylalanine hydroxylase; RESP, responders; S‐RESP, slow responders.

Ranking variants by tested carriers when acting as the milder allele identified alleles with high and low responder rates (Figure 6, Table 4, and Table S2), showing that BH4 responsiveness depends on genotype context, including the severity of the allele in trans (e.g., c.1222C>T|c.1241A>G (82.8%) in Table S1).

**TABLE 4:** Top variants associated with BH4 responsiveness when acting as the milder allele (top 20 by tested carriers).

Variant	Carriers where variant is mild	Label	Responder rate when mild (%)
c.782G>A	452	Intermediate/variable	34.3
c.1222C>T	388	Associated with N‐RESP (as mild allele)	2.1
c.1241A>G	340	Associated with RESP (as mild allele)	89.1
c.1066‐11G>A	312	Associated with N‐RESP (as mild allele)	2.9
c.143T>C	274	Intermediate/variable	62.8
c.194T>C	181	Associated with N‐RESP (as mild allele)	11.6
c.1169A>G	176	Associated with RESP (as mild allele)	100
c.1208C>T	156	Associated with RESP (as mild allele)	100
c.1162G>A	147	Intermediate/variable	52.4
c.842C>T	145	Associated with N‐RESP (as mild allele)	1.4
c.898G>T	127	Associated with RESP (as mild allele)	100
c.728G>A	126	Associated with N‐RESP (as mild allele)	0.8
c.721C>T	123	Associated with RESP (as mild allele)	87.8
c.1315+1G>A	92	Associated with N‐RESP (as mild allele)	4.3
c.1042C>G	90	Intermediate/variable	32.2
c.473G>A	84	Associated with N‐RESP (as mild allele)	2.4
c.204A>T	82	Intermediate/variable	70.7
c.838G>A	60	Associated with N‐RESP (as mild allele)	1.7
c.441+5G>T	54	Associated with N‐RESP (as mild allele)	0

## 4 Discussion

Using a large multicenter cohort from the BIOPKU resource, we quantified strong genotype dependence of BH4 responsiveness across the clinical spectrum of PAH deficiency. Among tested individuals, the proportion classified as responders (RESP+S‐RESP) was highest in mild hyperphenylalaninemia (99.3%), intermediate in mild PKU (73.2%), and lowest in classic PKU (6.0%) (Figure 1 and Table 1). This distribution is consistent with the expected relationship between residual PAH activity and pharmacologic responsiveness to BH4.

APV captures this relationship in a clinically interpretable milder‐versus‐severer allele framework. When the “milder” allele was defined as the allele with the higher APV, responder probability increased progressively across milder‐allele APV strata (Figure 2 and Table 2), while increasing 0‐allele burden was associated with reduced BH4 responsiveness (Figure 3). These findings support and extend earlier APV‐based frameworks for predicting phenotype and BH4 responsiveness from genotype and reinforce the practical observation that, in compound heterozygotes, the allele with greater residual function is usually the stronger determinant of both metabolic severity and treatment response [9, 10]. This pattern was also consistent across VEP‐derived genotype functional classes (Figure 4).

In prediction analyses designed to avoid information leakage between identical genotypes, APV alone provided high discrimination of responders versus nonresponders, consistent with prior BIOPKU‐derived work. Integrating functional consequence annotation derived from Ensembl VEP yielded only a modest increase in discrimination (AUROC 0.945–0.950 in genotype‐held‐out validation; Figure 5 and Table 3). Accordingly, we do not interpret this small AUROC difference as evidence of substantial standalone clinical gain. Rather, the principal practical advantage of the integrated approach is improved coverage and interpretability for alleles that are rare, previously uncharacterized, or insufficiently represented in APV‐based frameworks. The observation is consistent with earlier work combining genotype databases with in silico variant‐effect predictors for BH4 response inference [11] but is implemented here using standardized VEP‐derived consequence categories aligned with current annotation practice.

Our primary objective was generalizable prediction for real‐world genotypes, where functional annotation fields are frequently missing by design for non‐applicable variant classes. A conservative imputation strategy and genotype‐held‐out validation reduce the risk that performance is driven by artifact or leakage and reflect expected performance in clinical deployment.

At the variant level, the most frequent variants acting as the milder allele showed distinct responder profiles (Table 4 and Table S2), emphasizing that BH4 responsiveness is not a property of single variants in isolation but of genotype context, including the severity of the allele in trans and possible modifying influences. This supports current best‐practice recommendations that genotype‐based prioritization should complement, rather than replace, standardized BH4 responsiveness testing when clinically indicated.

**FIGURE 7:**
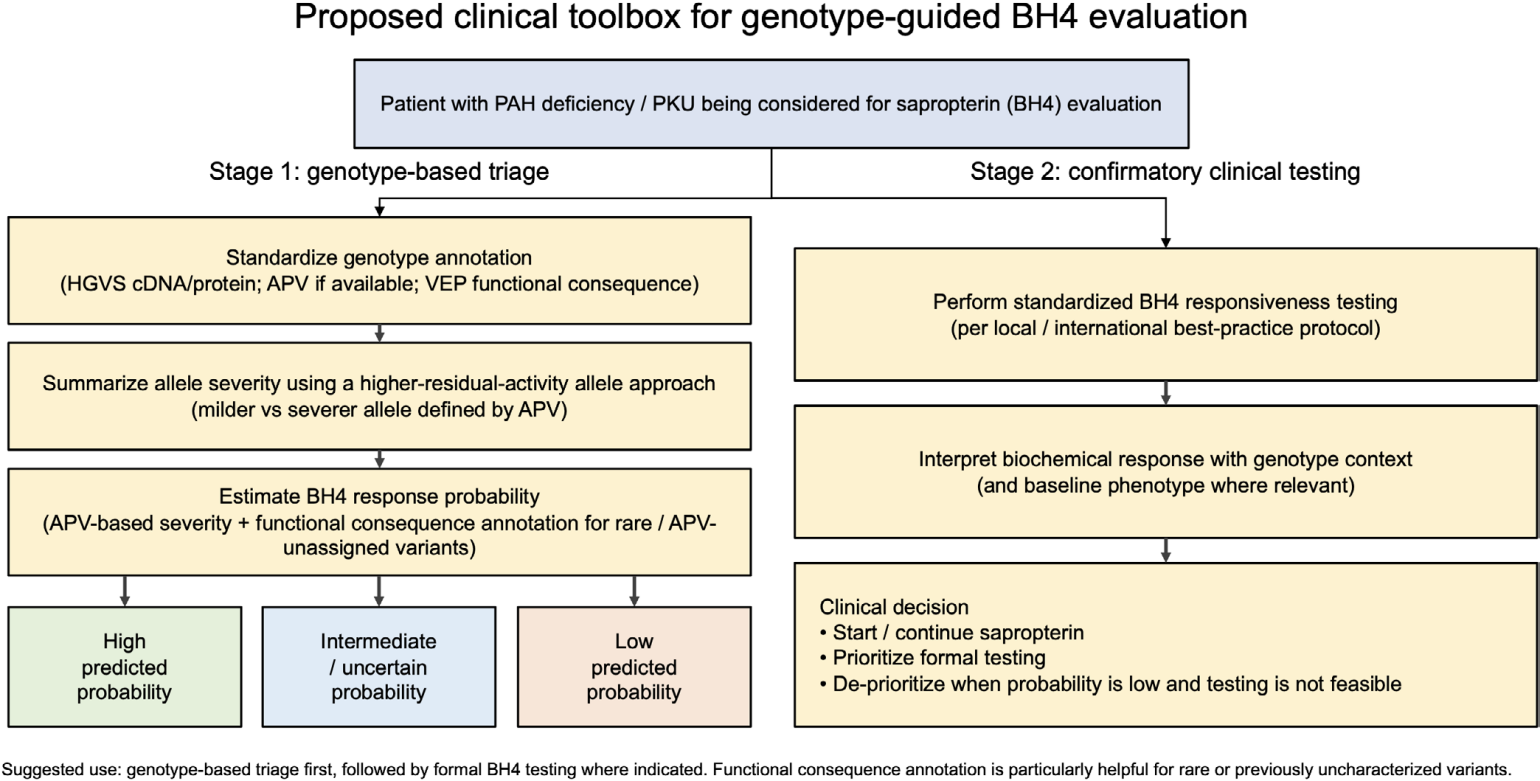
Proposed clinical toolbox for genotype‐guided BH4 evaluation. Schematic two‐stage workflow for clinical evaluation of BH4 responsiveness in PAH deficiency. In stage 1, genotype‐based triage is performed by standardizing variant annotation, summarizing allele severity using an APV‐based milder‐versus‐severer allele assessment, and estimating the probability of BH4 responsiveness with incorporation of functional consequence annotation for rare or APV‐unassigned variants. This triage step classifies patients into high, intermediate/uncertain, or low predicted probability of BH4 responsiveness. In stage 2, patients undergo standardized BH4 responsiveness testing and integrated clinical interpretation, leading to treatment decisions regarding sapropterin use and prioritization of formal testing. The figure is intended as a practical clinical framework derived from the present findings and not as a substitute for standardized BH4 response testing where clinically indicated. APV, allelic phenotype value; BH4, tetrahydrobiopterin; PAH, phenylalanine hydroxylase; PKU, phenylketonuria; VEP, variant effect predictor.

Clinically, these findings support a two‐stage approach: first, use APV‐based severity estimates to identify likely responders and clear nonresponders; second, refine the intermediate zone by incorporating functional consequence features that indicate whether the genotype plausibly retains chaperone‐rescuable PAH activity (Figure 7). Such stratification may improve counseling and help prioritize BH4 loading tests and longer‐term sapropterin trials using established response criteria and consensus testing protocols [6, 8]. In parallel, PAH activity landscapes derived from experimental systems may provide a complementary functional perspective for common genotypes and help visualize genotype‐specific enzymatic behavior under different metabolic or therapeutic conditions, thereby supporting more individualized treatment strategies [12]. Broader clinical experience with sapropterin across PAH deficiency and BH4 deficiency has been reviewed elsewhere [13].

Limitations of the study include heterogeneity in BH4 testing protocols and follow‐up across contributing centers, possible misclassification of slow responders, and incomplete annotation for rare or complex alleles. In silico functional predictors are imperfect surrogates for residual activity and cannot substitute for direct biochemical or cellular functional assays. In addition, prediction models were trained on tested individuals; therefore, extrapolation to untested patients should be interpreted as probabilistic prioritization rather than definitive classification.

Overall, APV provides a robust backbone for genotype‐based prediction of BH4 responsiveness, and the addition of standardized functional consequence annotation offers complementary information that modestly improves discrimination while increasing biological interpretability. Together, these approaches provide a practical path toward more consistent, genotype‐informed assessment of BH4 responsiveness in PAH deficiency.

## Funding

The authors have nothing to report.

## Ethics Statement

This study used de‐identified data retrieved from the BIOPKU database under authorization granted to the participating investigators and in accordance with BIOPKU data‐governance policies. The analysis was performed on de‐identified retrospective data and did not require additional ethics approval.

## Conflicts of Interest

The authors declare no conflicts of interest.

## Supporting information


**Figure S1:** Confusion matrix for the APV plus functional consequence annotation model.

**Table S1:** Top 20 genotypes by number of tested individuals with BH4 responder rate.
**Table S2:** Top 20 variants by carriers when acting as the milder allele, with BH4 responder rate.

## Data Availability

The data that support the findings of this study are available from the corresponding author upon reasonable request.
