# Supplementary figures and images for: Integrating Functional Consequence Annotation With PAH Allelic Phenotype Values Refines Prediction of Tetrahydrobiopterin Responsiveness
Source: J Inherit Metab Dis. 2026 Jun 21;49(4):e70220. doi: 10.1002/jimd.70220 (PMC13283760; doi:10.1002/jimd.70220)

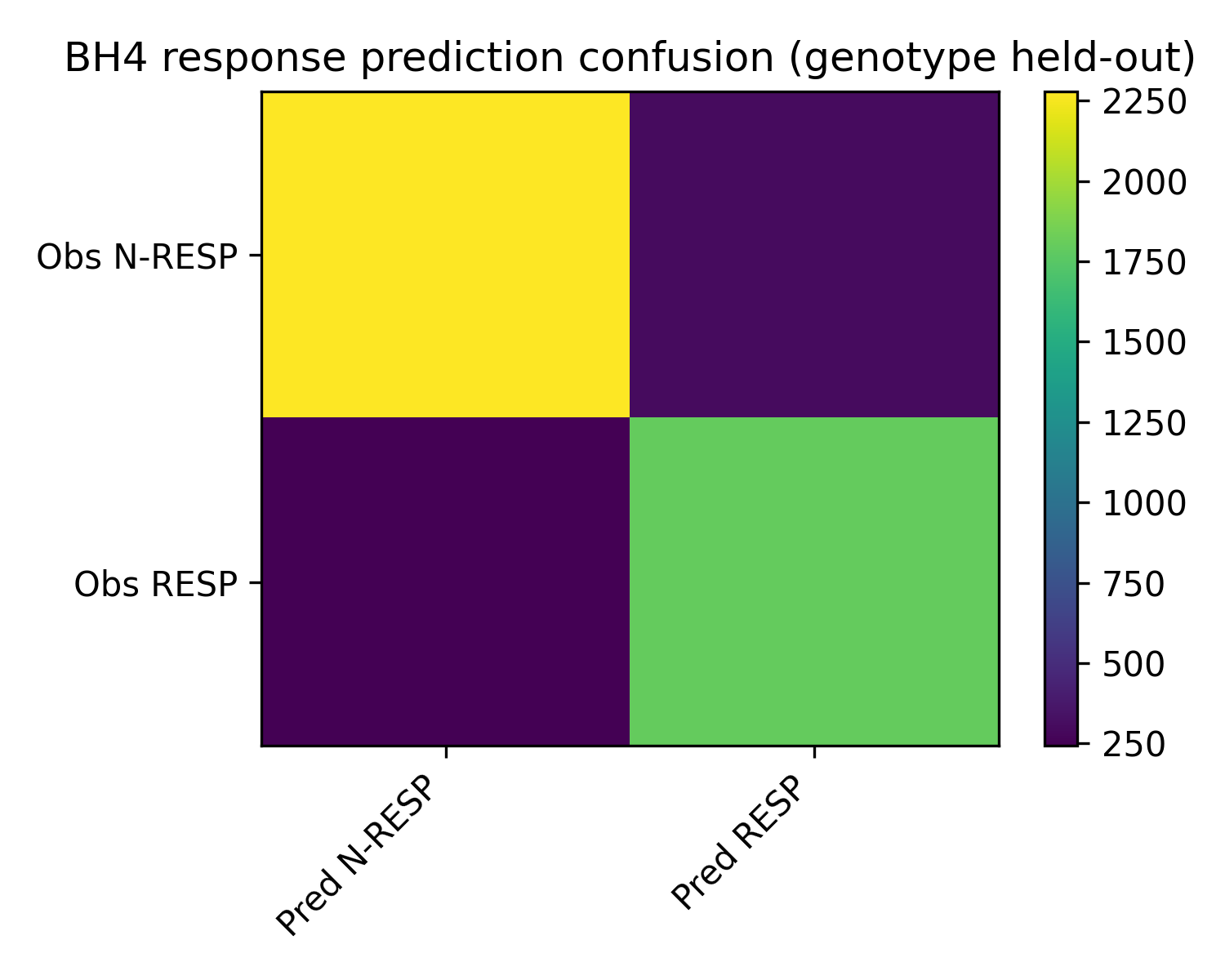

Supplement: Supplementary file 1 — Figure S1: Confusion matrix for the APV plus functional consequence annotation model. [file JIMD-49-0-s002.png]
