# Supplementary material for: Integrating Functional Consequence Annotation With PAH Allelic Phenotype Values Refines Prediction of Tetrahydrobiopterin Responsiveness
Source: J Inherit Metab Dis. 2026 Jun 21;49(4):e70220. doi: 10.1002/jimd.70220 (PMC13283760; doi:10.1002/jimd.70220)
Supplement: Supplementary file 2 — Table S1: Top 20 genotypes by number of tested individuals with BH4 responder rate. Table S2: Top 20 variants by carriers when acting as the milder allele, with BH4 responder rate. [file JIMD-49-0-s001.docx]

**Supplementary Tables**

**Supplementary Table S1. Top 20 genotypes by number of tested individuals with BH4 responder rate.**

| **Genotypes** | **Tested (n)** | **Responder (n)** | **Responder rate (%)** |
| --- | --- | --- | --- |
| c.1066-11G>A\|c.1066-11G>A | 126 | 3 | 2.4 |
| c.782G>A\|c.782G>A | 104 | 79 | 76.0 |
| c.1222C>T\|c.1222C>T | 101 | 1 | 1.0 |
| c.143T>C\|c.143T>C | 64 | 54 | 84.4 |
| c.1222C>T\|c.1241A>G | 58 | 48 | 82.8 |
| c.1066-11G>A\|c.782G>A | 54 | 3 | 5.6 |
| c.842C>T\|c.842C>T | 48 | 0 | 0.0 |
| c.1222C>T\|c.782G>A | 45 | 3 | 6.7 |
| c.1222C>T\|c.1315+1G>A | 42 | 0 | 0.0 |
| c.1222C>T\|c.143T>C | 36 | 11 | 30.6 |
| c.1066-11G>A\|c.1241A>G | 34 | 29 | 85.3 |
| c.143T>C\|c.782G>A | 34 | 27 | 79.4 |
| c.838G>A\|c.838G>A | 32 | 0 | 0.0 |
| c.1241A>G\|c.1315+1G>A | 32 | 30 | 93.8 |
| c.1315+1G>A\|c.782G>A | 30 | 1 | 3.3 |
| c.1066-11G>A\|c.143T>C | 27 | 17 | 63.0 |
| c.611A>G\|c.728G>A | 27 | 0 | 0.0 |
| c.1066-11G>A\|c.1222C>T | 26 | 1 | 3.8 |
| c.1169A>G\|c.1222C>T | 26 | 26 | 100.0 |
| c.1222C>T\|c.473G>A | 25 | 0 | 0.0 |

**Supplementary Table S2. Top 20 variants by carriers when acting as the milder allele, with BH4 responder rate.**

| Variant | Carriers where variant is mild | Label | Responder rate when mild (%)% |
| --- | --- | --- | --- |
| c.782G>A | 452 | Intermediate/variable | 34.3 |
| c.1222C>T | 388 | Associated with N-RESP (as mild allele) | 2.1 |
| c.1241A>G | 340 | Associated with RESP (as mild allele) | 89.1 |
| c.1066-11G>A | 312 | Associated with N-RESP (as mild allele) | 2.9 |
| c.143T>C | 274 | Intermediate/variable | 62.8 |
| c.194T>C | 181 | Associated with N-RESP (as mild allele) | 11.6 |
| c.1169A>G | 176 | Associated with RESP (as mild allele) | 100.0 |
| c.1208C>T | 156 | Associated with RESP (as mild allele) | 100.0 |
| c.1162G>A | 147 | Intermediate/variable | 52.4 |
| c.842C>T | 145 | Associated with N-RESP (as mild allele) | 1.4 |
| c.898G>T | 127 | Associated with RESP (as mild allele) | 100.0 |
| c.728G>A | 126 | Associated with N-RESP (as mild allele) | 0.8 |
| c.721C>T | 123 | Associated with RESP (as mild allele) | 87.8 |
| c.1315+1G>A | 92 | Associated with N-RESP (as mild allele) | 4.3 |
| c.1042C>G | 90 | Intermediate/variable | 32.2 |
| c.473G>A | 84 | Associated with N-RESP (as mild allele) | 2.4 |
| c.204A>T | 82 | Intermediate/variable | 70.7 |
| c.838G>A | 60 | Associated with N-RESP (as mild allele) | 1.7 |
| c.441+5G>T | 54 | Associated with N-RESP (as mild allele) | 0.0 |
| c.722G>A | 53 | Intermediate/variable | 77.4 |
